# The Airway Volume Related to the Maxillo-Mandibular Position Using 3D Analysis

**DOI:** 10.1155/2021/6670191

**Published:** 2021-06-21

**Authors:** Víctor Ravelo, Gabriela Olate, Gonzalo Muñoz, Márcio de Moraes, Sergio Olate

**Affiliations:** ^1^Centre of Excellence in Morphological and Surgical Studies, University of La Frontera, Chile; ^2^Division of Oral and Maxillofacial Surgery, Piracicaba Dental School, State University of Campinas, Brazil; ^3^Division of Oral, Facial and Maxillofacial Surgery, Dental School, University of La Frontera, Chile

## Abstract

**Objective:**

The aim of this research was to compare three cephalometric analyses and their correlation with the airway volume in subjects with different skeletal classes using 2D and 3D images. *Study Design*. Cross-sectional descriptive study. *Material and Method*. Steiner, McNamara, and Ricketts analyses and the airway volume were compared in 115 subjects who were candidates for orthognathic surgery under diagnosis using cone beam computed tomography (CBCT); 46 males (40%) and 69 females (60%) were included. The sagittal positions of the maxilla and mandible, the angulation of the mandibular plane, the sagittal positions of the upper and lower incisors, measurements of the largest or shortest airway area, and the volume were compared using Spearman's test considering a *p* value < 0.05.

**Results:**

Differences were observed between the Steiner and McNamara techniques for the sagittal position of the maxilla (*p* = 0.01). For mandibular angulation, there was a greater difference between values for Steiner and Ricketts techniques (*p* = 0.001). In the upper incisor, the results for McNamara and Ricketts techniques were significantly different (*p* = 0.004). Analysing the airway, subjects with a class II skeletal pattern had a smaller volume than those with a class III pattern (*p* = 0.034).

**Conclusion:**

It may be concluded that skeletal class II patients have a significantly smaller airway volume than class III patients. The skeletal parameter does not always relate to the airway volume; however, the high mandibular angle could be related to the airway conditions.

## 1. Introduction

Some aetiological factors can modify the development of the face and airway system. Variations in some anatomical structures can produce deformities that affect the aesthetics and function of the craniomaxillofacial complex [1, 2].

The most used analyses in orthodontics and craniomaxillofacial surgery are cephalometric and panoramic X-rays, transcranial projections, and cone beam computed tomography (CBCT). The advantages of CBCT include the low dose of radiation, standardized positioning of the patient, high-quality images without the superposition of structures, and a better cost-benefit ratio [3, 4].

Cephalometric analyses enable the study of the tooth positions, facial growth patterns, anatomical deformities, and hard and soft tissue analysis, as well as orthodontic, orthosurgical, and surgical treatment planning [5]. Some cephalometric values are related to an increase or decrease in mandibular angles, sagittal differences in the maxilla and mandible, and differences in facial height, involving functional changes in the oral and nasal systems [6] together with occlusal alterations [7]. Furthermore, changes in the airway can also be observed with these morphological characteristics [8].

Although cephalometric images do not always give precise data about the morphology of the airways, they allow the definition of skeletal patterns that can be related to oral and facial characteristics and related to the size or morphology of the airway [9–11]. In some skeletal patterns, differences are noted between cephalometric analyses, which can be influenced by the landmarks used in each technique, the position of the head, the vertical and horizontal positions of the hard and soft tissues, and others [11].

The aim of this research was to compare three cephalometric techniques and the airway volume in subjects with different skeletal classes using two-dimensional (2D) images for cephalometry and 3-dimensional (3D) images for analysing the airway volume.

## 2. Materials and Methods

A comparative analysis was performed using Steiner, McNamara, and Ricketts cephalometric analyses in 115 subjects who were candidates for orthognathic surgery and was related to 3D analysis of the airway. CBCT was used in the facial diagnosis prior to orthodontic treatment. The participants signed an informed consent and agreed voluntarily to the study. The research protected the participants' integrity, respected the Declaration of Helsinki, and was approved by protocol 027-17.

Males and females with class II or class III dentofacial deformity were included (following the Steiner criteria, class II: ANB > 4° and class III: ANB < 0°). Subjects with a history of facial trauma or facial syndromes or who had had previous facial surgery were excluded, as were subjects with evident facial asymmetries defined by a chin deviation of more than 5 mm from the facial midline.

**Figure 1:**
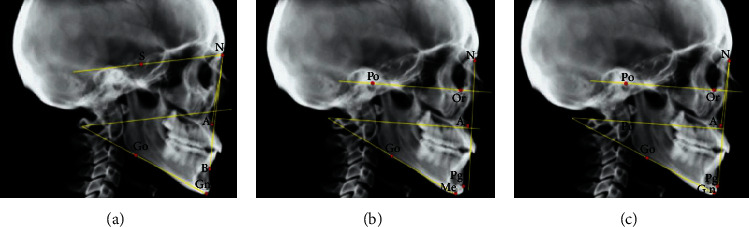
Cephalometric points used in the Steiner, McNamara, and Ricketts techniques. (a) Steiner analysis: the anteroposterior positions of the maxilla (S-Na) and mandible (S-N-B) and angulation of the mandibular plane (S-N/Go-Ng) were determined. (b) McNamara analysis: the anteroposterior positions of the maxilla (Na-perpendicular-A) and mandible (Na-perpendicular-Pog) and angulation of the mandibular plane (Po-Or/Go-Me) were determined. (c) Ricketts analysis: the anteroposterior positions of the maxilla (Frankfort Plane-Na-A) and mandible (Frankfort Plane-Na-Pog) and angulation of the mandibular plane (Po-Or/Go-Gn) were determined.

The three cephalometric studies were conducted on each subject (Figure 1), comparing the sagittal positions of the maxilla and the mandible, the angulation of the mandibular plane, and the positions of the upper and lower incisors as shown in Table 1.

### 2.1. Positions of the Maxilla and Mandible

The Steiner technique included the S-Na line (line formed between the landmark located at the geometric centre of the sella turcica and the most anterior point of the frontonasal suture) as a reference plane. In order to determine the sagittal position of the maxilla, the S-Na plane was related to the A point (landmark in the most anterior region of the anterior maxillary concavity), forming the S-Na angle (normality parameter 82 ± 2°). For the mandibular position, the S-Na plane and B point (landmark in the most anterior region of the anterior mandibular concavity) were used, forming the SNB angle (normality parameter 80 ± 2°).

In both the McNamara and Ricketts techniques, the Frankfort plane was used, formed by the Po-Or landmarks (porion: highest edge of the external auditory canal and orbital: lowest point of the cranial orbit, respectively) and traced a perpendicular through the Na point. In order to observe the position of the maxilla for McNamara analysis, the distance was measured from the A point to the perpendicular (normality parameter 1 mm) and the Ricketts analysis measured the angulation formed by the Po-Or and Na-A point lines (normality parameter 90°). To determine the sagittal position of the mandible for McNamara analysis, the distance was measured from the Pog (pogonion: most anterior point on the anterior edge of the mandibular symphysis) to the perpendicular (normality parameter 2 ± 4 mm). The Ricketts analysis included the angulation formed by the Po-Or and Na-Pog lines (normality parameter 87 ± 90°).

### 2.2. Angulation of the Mandibular Plane

To obtain the angulation of the mandibular plane using the Steiner and Ricketts techniques, the intersection of the S-Na and Go-Gn lines (Go: point located at the most posterior and lowest part of the mandibular angle; Gn: most anterior and lowest landmark of the anterior edge of the mandibular symphysis, normally located between the chin and the pogonion) was used. For the Steiner analysis, a normality parameter of 36° was used and for the Ricketts analysis a normality parameter of 26 ± 4° was used. For the McNamara technique, the intersection of the Po-Or and Go-Me lines (Me: chin, lowest point of the mandibular symphysis) was used, with a normality parameter of 25°.

### 2.3. Incisor Inclination in the Maxilla and Mandible

In the 2D images, the corresponding traces were made to determine the positions of the upper and lower incisors. In the Ricketts analysis, a vertical was determined from the A Point to the Pog to establish the position of the incisors in the maxilla and the mandible. The distance was measured from the vertical to the most buccal point of the upper incisor (normality parameter 3.5 mm) and lower incisor (normality parameter 1 mm).

To determine the position of the upper incisor, in both the Steiner and McNamara analyses, a vertical was determined between the Na point and A point. For the Steiner analysis, the distance was measured between the vertical and the incisor edge, and for the McNamara analysis the, distance was measured from the vertical to the most buccal point of the incisor. Finally, to define the incisor position in the mandible, the Steiner analysis determined a vertical from the Na point to the B point and the distance was measured between the vertical and the incisor edge (normality parameter 4 mm). In the McNamara analysis, a vertical was determined between the Na and Pog points and the distance was measured between the vertical and the buccal surface of the incisor (normality parameter 3 mm).

### 2.4. Airway

For analysis of the airway, a CBCT image acquired with a NewTom 3D Tomograph, model VGi EVO (Verona, Italy), 24 × 19 cm window, was used, with an exposure time of 15 s at 110 kV and 8 mA. The images were obtained by two specialist technicians with 5 years of experience. The patient was immobilized, vertically, with the lips at rest and without forcing a muscle position. Once the image was obtained, the measurement was analysed using NewTom NNT (Imola, Italy) software by a trained specialist. The software created an algorithm to establish the total airway volume, including the minimum and maximum areas.

The landmarks used for this research were (1) anterior: posterior nasal spine in the sagittal plane and choanae in the axial plane, (2) posterior: posterior wall of the pharynx, (3) upper: highest point of the nasopharynx, and (4) lower: under the hyoid bone at the level of the lower edge of the C4 vertebral body.

The measurements were taken by the same observer on two different occasions with a 2-week interval between them. The results were analysed by concordance analysis, obtaining a kappa value of 0.88.

A 95% confidence interval was used to measure the agreement of the maxillary and mandibular positions among the techniques. In addition, Spearman's test was performed to determine the correlation between the variables and their relation to the airway volume. A *p* value of <0.05 was used to determine significant difference.

## 3. Results

One hundred and fifteen subjects were included, 46 (40%) male and 69 (60%) female, ranging in age from 18 to 55 years. Comparing the Steiner, McNamara, and Ricketts analyses to define the sagittal position of the maxilla, it was observed that the Steiner analysis presented a greater prevalence of subjects with maxillary retrognathism (42.60%). On the other hand, the McNamara analysis presented a greater prevalence of individuals with maxillary prognathism (54.78%), followed by the Ricketts analysis (53.04%). Finally, both the Steiner and Ricketts analyses had a similar prevalence (33.91% and 36.52%) of subjects with the maxilla in a normal position.

At the mandibular level, the Steiner analysis showed a larger number of subjects with retrognathism than for the other two techniques (50.43%). The McNamara and Ricketts analyses had identical values to determine mandibular protrusion (41.73%), and both analyses had a similar number of subjects with the mandible in the normal position as shown in Table 2.

In terms of mandibular angulation, a greater prevalence was obtained using the Ricketts technique over the other two techniques for subjects with normal (29.57%) and reduced (convergent) angulation (50.43%) of the mandible. Finally, a larger number of individuals presented mandibular divergence values with the Steiner and McNamara techniques than the Ricketts technique as shown in Table 3.

For the position of the upper incisor, the Steiner technique presented the greatest prevalence of protruded incisors (56.52%) followed by the McNamara and Ricketts techniques (46.08% and 41.73%, respectively). The Ricketts technique presented the largest number of individuals with retruded incisors, followed by the McNamara and Steiner techniques (36.52%, 13.04%, and 20.87%). Finally, the Steiner and McNamara techniques presented a similar number of subjects with normally positioned maxillary incisors, unlike the Ricketts technique, which had a smaller average for this parameter. On the other hand, at the mandibular level, the Ricketts technique had the highest number of individuals with protruded incisors (83.47%); values obtained for the McNamara and Steiner techniques were similar to each other and less than those obtained by Ricketts analysis (13.91% and 12.17%). The Steiner technique had a larger number of individuals with lower incisors in a normal position (25.22%) followed by the McNamara technique, which had a higher number than that obtained with the Ricketts technique. Finally, the Ricketts technique had a smaller number of individuals with retruded mandibular incisors than those for the Steiner and McNamara techniques as shown in Table 4.

In more specific analyses, the McNamara study was the only one that provided some significant analysis of variables for studies of the airway, indicating that the minimum airway area is correlated with mandibular angulation (*p* = 0.04) and the total airway volume is related significantly to the position of the maxilla (*p* = 0.04). No differences were observed between the smallest or largest airway area or total airway volume using other variables. It was evidenced by the three cephalometry techniques that divergent mandibular angulation showed a lower airway volume when comparing it to convergent mandibular angulation, showing differences of 17 ± 20 mm^2^ in the minimum area and 18 ± 52 mm^2^ in the maximum area with no statistical differences as shown in Table 5.

**Figure 2:**
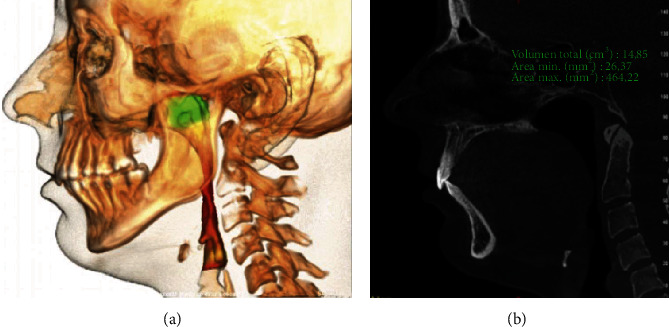
(a) 3D projection of the airway in subjects with class II skeletal deformity. (b) Lateral projection of the same subject.

**Figure 3:**
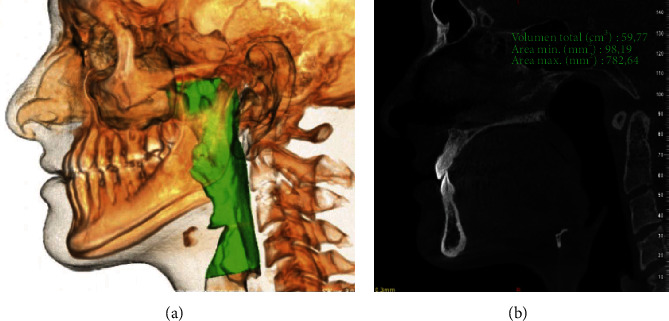
(a) 3D projection of the airway in subjects with class III skeletal deformity. (b) Lateral projection of the same subject.

For the airway analysis, it was observed that the volume was significantly greater in class III subjects (*p* = 0.034) (Figures 2 and 3). Observing the airway values independently, it was noted on all levels that subjects with class II characteristics presented lower values than class III subjects.

Similarly, Spearman's test revealed a lack of agreement in the sagittal analysis of the maxilla between the Steiner and McNamara techniques (*p* = 0.01). For mandibular angulation, the Steiner and Ricketts techniques presented the greatest discrepancy in their values (*p* = 0.001), and for maxillary incisor position, only the McNamara and Ricketts techniques presented statistically significant differences (*p* = 0.004).

## 4. Discussion

Studies using 2D images have made a great contribution to analysis and diagnosis of subjects with dental and facial deformities. Although 2D lateral X-rays are an important tool for skeletal analyses, they have a series of limitations, such as the superposition of structures and the absence of Hounsfield correspondence in soft tissues, which make it difficult to visualize structures like the airway [12].

It has been reported [13] that when comparing 2D and 3D images, the latter provide more reliable and accurate dimensions than conventional cephalometry in hard tissues and soft tissues and in measurements of airways (volume). In this sense, Sear et al. [14] showed no correlation to define the airway in cephalometry.

When comparing the Steiner, McNamara, and Ricketts analyses to determine skeletal characteristics, differences in the measurements of normality and abnormality were observed. In the sagittal analysis, only the McNamara and Ricketts techniques had similar percentages for the maxillary and mandibular position, which may be related to the similar landmarks used in measurements. Cevidanes et al. [15] and Ruellas et al. [16] indicated that to obtain reliable analyses, the images must be acquired in a standardized way, with the head in a natural position and with stable reference planes. In addition, a system of coordinates must be established to obtain a standardized way, which was done in the present research.

On the other hand, when conducting Steiner analysis, there are differences in the measurement of the sella turcica in adolescents and young adults due to its calcification, which makes it difficult to locate and determine the maxillary and mandibular sagittal positions at the same time [17]. Similar conclusions have been drawn from other research in which it was observed that clinical match is not consistent in determining the maxillary and mandibular positions [18, 19]; for that reason, the results for these angles should be complemented with another method of analysis, as confirmed in our research.

Using a superposition of several cephalometric techniques, Lenza et al. [20] concluded that both Steiner and Ricketts analyses present similar accuracy when doing the tracing, which contrasts with our results, because we observed the greatest differences between their results. Grogger et al. [21] showed that landmarks and the cephalometric tracings of true lines can generate the most errors when determining sagittal positions or skeletal inclinations. Therefore, standardization and a regular definition of the landmarks and planes to be used are the most important factors for obtaining comparable results between patients. In this research, in the analysis of the mandibular angle, Steiner and Ricketts analyses present similar landmarks but the results for these were not similar due to the difference in normality values that each author included.

In the analysis of the incisor position, there were similar percentages for each diagnosis. This is related to the cephalometric landmarks used in each analysis, since they all take measurements at points A and/or B/Pog; in addition, the three techniques had similar normality values based on millimetres, which permit greater proximity of the results.

Previous studies mentioned that cephalometric measures are not applicable to subjects from different ethnic groups, since they have morphological characteristics different to those of Caucasian subjects mainly in the mandibular sagittal position, which is on average retruded, increasing mandibular angulations [22, 23].

In this sense, Gu et al. [24] compared the craniofacial characteristics of Chinese and Caucasian subjects with normal occlusions and balanced facial biotypes, concluding that there were significant differences between hard and soft tissues in the groups. Similar studies agree that significant differences in soft tissue, labial inclinations, and incisor position are observed when measurements are taken with cephalometric techniques in patients from different ethnic groups [25, 26].

In the cephalometric analyses of the airway volume, it was observed that subjects with a class II deformity had a smaller airway volume than subjects with a class III deformity. However, in some class III subjects, these values were similar to those for subjects in class II. The results for 3D analysis of the airway also showed that these differences were statistically significant when the subjects were grouped. Nevertheless, when analysing the data individually, it was noted that values in some subjects in facial class III were considerably lower and similar to those for subjects in facial class II. In their study, Cheng et al. [27] reported that class III subjects have a greater airway volume than class I and II patients both before and after orthognathic surgery.

Pavoni et al. [28] showed in their study that early mandibular advancement with an orthodontic appliance is a good treatment alternative in patients with a class II skeletal deformity. When used during growth, patients with these devices showed significant improvements in respiratory symptoms, related to a significant decrease of ANB angle and overjet, which confirms our findings that patients with a class III skeletal malocclusion (lower values of ANB and overjet) have a greater airway volume. A long-term study led by Pavoni et al. [29] also showed that the changes produced by early mandibular advancement with these devices are maintained in the long term until after puberty.

Cretella Lombardo et al. [30] analysed the upper airway in class III patients treated with maxillary disjunction and a frontal traction mask compared with untreated patients. Their results showed that there were significant changes in the airway in treated patients compared with nontreated patients. Their results suggest that both horizontal and anteroposterior augmentation (expansion and reverse traction) of the maxilla would be associated with an increase in the airway volume, which confirms our findings in terms of airway analysis.

Oliveira et al. [31] indicated that in CBCT, the image time for acquisition can vary between 20 and 38 s, which can be considered too long to ask the individual not to breathe. From this point of view, the airway volume on the 3D image can be influenced by muscle, postural, and morphological movements typical of the breathing cycle and must be considered in future analyses. Thus, the only value that showed a significant relation to the airway volume was mandibular angulation taken in the McNamara analysis, which could indicate regularity as a variable in cephalometric diagnosis for the airway. However, Cillo et al. [32] mentioned that craniofacial alterations are not always related to a reduction of the airway volume. Therefore, together with facial deformities, there are other variables such as subjects' BMI (body mass index) and height that influence the airway volume [33]. Changes in the position and craniocervical angle modify the airway volume and the position of the hyoid bone, which is an important variable to be considered when analysing the data [34, 35].

## 5. Conclusions

It may be concluded that there is no relation in the cephalometric analyses and 3D airway in terms of diagnosis; class II patients tend to show a smaller airway volume than class III patients. In addition, the skeletal parameter does not always relate to the airway volume; however, a high mandibular angle could be related to the airway conditions.

Other 3D studies that analyse morphological changes in the mandible and maxilla at different ages should be carried out to understand how these structures are related to the airway.

## Figures and Tables

**Table 1:** Comparative picture of the cephalometric points and normality parameters used.

	Steiner	*n*	McNamara	*n*	Ricketts	*n*
Mx position	S-Na-A point	82°	Po-Or/Na-perpendicular	1 mm	Po-Or/Na-A point	90°
Mn position	S-Na-B point	80°	Po-Or/Na-perpendicular	2 ± 4 mm	Po-Or/Na-Pog	87 ± 90°
Angulation Mn plane	S-Na/Go-Gn	32°	Po-Or/Go-Me	25°	Po-Or/Go-Gn	26 ± 4°
Mx incisor inclination	Na-A point/incisor edge	4 mm	Na-A point/up. buccal	4 mm	A point-Pog/up. vestibular	3.5 mm
Mn incisor inclination	Na-B point/incisor edge	4 mm	Na-Pog/up. vestibular	3 mm	A point-Pog/up. vestibular	1 mm

Mn: mandible; Mx: maxilla; S: point located at the geometric centre of the sella turcica; Na: most anterior point of the frontonasal suture; A point: point located in the most anterior region of the anterior maxillary concavity; B point: point located in the most anterior region of the anterior mandibular concavity; Go: point located in the most posterior and lowest parts of the mandibular angle; Gn: most anterior and lowest points of the anterior edge of the mandibular symphysis, normally located between the menton and pogonion; Me: lowest point of the mandibular symphysis; Po: highest point of the external auditory canal; Or: lowest point of the orbit; Pog: most anterior point on the anterior edge of the mandibular symphysis.

**Table 2:** Anteroposterior distribution frequencies in the maxilla and mandible.

Position	Steiner technique		McNamara technique		Ricketts technique	
	*n*	%	*n*	%	*n*	%
Retruded maxilla	49	42.60	21	18.26	12	10.43
Protruded maxilla	27	23.47	63	54.78	61	53.04
Normal maxilla	39	33.91	31	26.95	42	36.52
Retruded mandible	58	50.43	39	33.91	34	29.56
Protruded mandible	45	39.13	48	41.73	48	41.73
Normal mandible	12	10.43	28	24.34	33	28.69

*n* corresponds to the number of individuals for each characteristic and is expressed in percentages in the column (%).

**Table 3:** Distribution frequency of the mandibular angulation for Steiner, McNamara, and Ricketts analyses.

Mandibular angulation	Steiner technique		McNamara technique		Ricketts technique	
	*n*	%	*n*	%	*n*	%
Divergent angulation	74	64.34%	73	63.47%	23	20.00%
Convergent angulation	34	29.56%	33	28.69%	58	50.43%
Normal angulation	7	6.08%	9	7.82%	34	29.57%

*n* corresponds to the number of individuals for each characteristic and is expressed in percentages in the column (%).

**Table 4:** Frequency distribution of the incisor inclination of the three techniques used in the maxilla and mandible.

Incisor inclination	Steiner technique		McNamara technique		Ricketts technique	
	*n*	%	*n*	%	*n*	%
Protruded maxillary incisor	65	56.52%	53	46.08%	48	41.73%
Retruded maxillary incisor	15	13.04%	24	20.87%	42	36.52%
Normal maxillary incisor	35	30.43%	38	33.04%	25	21.73%
Protruded mandibular incisor	72	62.61%	79	68.69%	96	83.47%
Retruded mandibular incisor	14	12.17%	16	13.91%	2	1.73%

*n* corresponds to the number of individuals for each characteristic and is expressed in percentages in the column (%).

**Table 5:** Correlation analysis between the Steiner, McNamara, and Ricketts cephalometric analyses and airway volume.

Cephalometry	*p* value	Minimum area (mm^2^)	Maximum area (mm^2^)	Total volume (mm^3^)
Steiner	Mx	*p* = 0.27	*p* = 0.10	*p* = 0.12
	Md	*p* = 0.30	*p* = 0.15	*p* = 0.15
	Md angulation	*p* = 0.26	*p* = 0.23	*p* = 0.44
McNamara	Mx	*p* = 0.39	*p* = 0.08	*p* = 0.04^∗^
	Md	*p* = 0.18	*p* = 0.35	*p* = 0.32
	Md angulation	*p* = 0.04^∗^	*p* = 0.14	*p* = 0.11
Ricketts	Mx	*p* = 0.34	*p* = 0.19	*p* = 0.43
	Md	*p* = 0.06	*p* = 0.39	*p* = 0.13
	Md angulation	*p* = 0.10	*p* = 0.38	*p* = 0.20

Values correspond to the statistical correlation between the type of cephalometric variable and the airway analysis variable. ^∗^Statistically significant difference.

## Data Availability

The statement will be provided by the author.

## Supplementary Materials

Supplementary Materials Table VI: anteroposterior positions of the maxilla and mandible were evaluated independently by the following the parameters set by Steiner. The values represent the airway volume of subjects who had a normal (I), retrognathic (II), or prognathic (III) maxilla (Mx), independent of the mandibular position. In the same way, the airway volume of subjects presenting a normal (I), retrognathic (II), or prognathic (III) mandibular position (Mn), independent of the maxillary position, was evaluated. The airway volume of subjects who presented convergence or divergence of the mandibular plane angulation and the airway volume of subjects with skeletal class (SC) II and III were also evaluated. Tables VII and VIII also show airway values associated with the anteroposterior positions of the maxilla and mandible but evaluated according to the McNamara and Ricketts parameters, respectively.
