# Supplementary material for: The Airway Volume Related to the Maxillo-Mandibular Position Using 3D Analysis
Source: Biomed Res Int. 2021 Jun 21;2021:6670191. doi: 10.1155/2021/6670191 (PMC8241511; doi:10.1155/2021/6670191)
Supplement: Supplementary Materials — Table VI: anteroposterior positions of the maxilla and mandible were evaluated independently by the following the parameters set by Steiner. The values represent the airway volume of subjects who had a normal (I), retrognathic (II), or prognathic (III) maxilla (Mx), independent of the mandibular position. In the same way, the airway volume of subjects presenting a normal (I), retrognathic (II), or prognathic (III) mandibular position (Mn), independent of the maxillary position, was evaluated. The airway volume of subjects who presented convergence or divergence of the mandibular plane angulation and the airway volume of subjects with skeletal class (SC) II and III were also evaluated. Tables VII and VIII also show airway values associated with the anteroposterior positions of the maxilla and mandible but evaluated according to the McNamara and Ricketts parameters, respectively. [file 6670191.f1.docx]

**Complementary Data**

**Table VI.**  Distribution of subjects studied according to Steiner cephalometric analysis and airway volume.

|  | Minimum area (mm^2^) | | Maximum area (mm2) | | Total volume (mm^3^) | |
| --- | --- | --- | --- | --- | --- | --- |
|  | x | SD | x | SD | x | SD |
| Mx I | 114.84 | 58.96 | 511.67 | 116.89 | 26.47 | 8.19 |
| Mx II | 115.35 | 60.26 | 555.20 | 129.27 | 28.74 | 8.19 |
| Mx III | 99.33 | 41.35 | 500.99 | 149.57 | 27.45 | 9.78 |
| Md I | 107.69 | 35.49 | 504.26 | 92.08 | 26.79 | 6.2 |
| Md II | 111.52 | 58.88 | 520.37 | 129.89 | 26.53 | 7.39 |
| Md III | 112.92 | 52.27 | 543.43 | 142.54 | 29.37 | 10.25 |
| Divergent angulation | 102.77 | 53.48 | 521.25 | 107.46 | 27.35 | 7.59 |
| Convergent angulation | 122.77 | 57.27 | 539.36 | 152.28 | 28.19 | 9.59 |
| SC II | 107.4 | 55.7 | 509.9 | 125 | 26.1 | 7.3 |
| SCIII | 117.8 | 54.8 | 556.5 | 134.9 | 30.2 | 9.6 |

**Note:** SC= skeletal class; Md= Mandible; Mx= Maxilla. I= normal; II= retruded; III= protruded. Values correspond to airway area and volume and are expressed in mm^2^ and mm^3^, with their respective standard deviation.

**Table VII.**  Distribution of subjects studied according to McNamara cephalometric analysis and airway volume.

|  | Minimum area (mm^2^) | | Maximum area (mm^2^) | | Total volume (mm^3^) | |
| --- | --- | --- | --- | --- | --- | --- |
|  | x | SD | x | SD | x | SD |
| Mx I | 102.60 | 58.98 | 500.02 | 131.94 | 27.10 | 7.87 |
| Mx II | 110.91 | 67 | 514.17 | 124.19 | 27.31 | 9.51 |
| Mx III | 115.93 | 50.51 | 545.83 | 132.61 | 28.07 | 8.69 |
| Md I | 102.83 | 54.51 | 546.29 | 98.05 | 27.86 | 8.89 |
| Md II | 110.53 | 55.20 | 509.26 | 125.07 | 26.40 | 7.25 |
| Md III | 116.06 | 58.48 | 551.45 | 149.48 | 29.77 | 10.16 |
| Divergent angulation | 101.85 | 55.26 | 515.21 | 116.86 | 27.54 | 8.25 |
| Convergent angulation | 118.3 | 57.49 | 557.08 | 156.01 | 28.34 | 10.05 |

**Note:** Md= Mandibula; Mx= Maxilla. I= normal; II= retruded; III= protruded. Values correspond to airway area and volume and are expressed in mm^2^ and mm^3^, with their respective standard deviation.

**Table VIII.**  Distribution of subjects studied according to Ricketts cephalometric analysis and airway volume.

|  | Minimum area (mm^2^) | | Maximum area (mm^2^) | | Total volume (mm^3^) | |
| --- | --- | --- | --- | --- | --- | --- |
|  | x | SD | x | SD | x | SD |
| Mx I | 113.40 | 56.66 | 515.98 | 115.96 | 27.67 | 10 |
| Mx II | 129.06 | 74.80 | 509.57 | 184.84 | 30.10 | 9.55 |
| Mx III | 106.59 | 51.24 | 539.35 | 130.81 | 27.19 | 7.28 |
| Md I | 98.32 | 56.19 | 527 | 112.24 | 27.06 | 7.65 |
| Md II | 115.09 | 52.94 | 503.84 | 138 | 25.63 | 6.25 |
| Md III | 117.84 | 57.32 | 544.89 | 138.70 | 29.53 | 10.20 |
| Divergent angulation | 104.97 | 51.31 | 490.62 | 131.72 | 27.06 | 6.48 |
| Convergent angulation | 122.05 | 57.19 | 542.04 | 139.80 | 28.43 | 9.65 |

**Note:** Md= Mandibula; Mx= Maxilla. I= normal; II= retruded; III= protruded. Values correspond to airway area and volume and are expressed in mm^2^ and mm^3^, with their respective standard deviation.
